# Damage-Associated Molecular Patterns and Myeloid-Derived Suppressor Cells in Bronchoalveolar Lavage Fluid in Chronic Obstructive Pulmonary Disease Patients

**DOI:** 10.1155/2019/9708769

**Published:** 2019-07-02

**Authors:** Beata Brajer-Luftmann, Agata Nowicka, Mariusz Kaczmarek, Magdalena Wyrzykiewicz, Senan Yasar, Tomasz Piorunek, Jan Sikora, Halina Batura-Gabryel

**Affiliations:** ^1^Department of Pulmonology, Allergology and Pulmonary Oncology, Poznan University of Medical Sciences, Szamarzewskiego 84 Street, 60-569 Poznan, Poland; ^2^Department of Clinical Immunology, Poznan University of Medical Sciences, Rokietnicka 5D Street, 60-806 Poznan, Poland

## Abstract

Myeloid-derived suppressor cells (MDSCs) are present in the human lung microenvironment, and they may be involved in the local inflammatory process in chronic obstructive pulmonary disease (COPD). Chronic inflammation in COPD may induce immunogenic cell death of structural airway cells, causing the release of damage-associated molecular patterns (DAMPs). DAMPs may activate the innate and adaptive immune system. The relationship between MDSCs and DAMPs in COPD is poorly described in the available literature. *Objectives*. (1) Assessment of MDSC percentage and DAMP concentration in bronchoalveolar lavage fluid (BALF) and peripheral blood. (2) Analysis of the relationship between MDSC percentage and chosen DAMPs. *Patients and Methods*. 30 COPD patients were included. Using monoclonal antibodies directly conjugated with fluorochromes in flow cytometry, MDSCs were assessed in BALF and peripheral blood. The concentration of DAMPs was estimated using sandwich ELISA. Using the Bradford method, the total protein concentrations were evaluated. *Results*. The percentage of MDSCs among MC in BALF correlated well with the concentration of defensin and heat shock protein 27. Assessing the percentage of MDSCs among all leukocytes in BALF, we revealed a significant correlation with the concentration of defensin, hyaluronic acid, and surfactant protein A. No dependencies occurred between DAMPs and MDSCs in peripheral blood. *Conclusion*. MDSCs and DAMPs occur in the COPD patient lung microenvironment. Significant correlations between them found in BALF may indicate their influence on the local inflammatory process in COPD. These relationships allow better understanding of the inflammatory process in COPD.

## 1. Introduction

Chronic obstructive pulmonary disease (COPD) is a significant cause of death in the world. Because of continued exposure to COPD risk factors and aging in the population, the COPD burden is projected to increase in the nearest future [1]. The inflammatory process in response to exposure to different environmental factors, including smoking, remains essential in the etiopathogenesis of this disease [2]. The chronic airway inflammation in COPD is characterized by the activation of the innate immune system, as defined by increased numbers of innate immune cells such as neutrophils, macrophages, natural killer cells, and mature dendritic cells in the lung tissue and airway lumen. Also, the adaptive immune system is activated in COPD, as defined by lung infiltration of CD8 T cells, B cells, and both the Th 17 and Th 1 types of CD4 T cells, along with a decrease in regulatory T cells in the airways [3]. These cells release soluble inflammatory agents, which interact with structural cells in the airways, lung parenchyma, and pulmonary vasculature. Various mediators that are increased in COPD patients attract the inflammatory cells from the circulation (chemoattractants) and enhance the inflammatory process (proinflammatory cytokines) [1].

Damage-associated molecular patterns (DAMPs) termed alarmins or “danger signals” are the endogenous particles secreted actively by viable cells and passively from dying cells. Their central role is to alert the organism to the negative consequence of tissue damage and initiate the repair process [4, 5]. Furthermore, DAMPs can also activate and mature cells of the innate as well as the adaptive immune system directly upon binding of pattern recognition receptors (PRRs), especially the Toll-like receptors (TLRs) [3, 6]. There is a close relationship between DAMPs and pathological processes like autoimmunity, chronic inflammation, and carcinogenesis [7, 8]. One of the hypotheses is that cigarette smoke-induced airway epithelial immunogenic cell death is followed by DAMP release and subsequent triggering of the innate and adaptive immune responses in COPD [9]. This theory is supported by a few new studies conducted in vitro and in vivo. They revealed that cigarette smoke extract (CSE) exposure on pulmonary epithelial cells can increase apoptosis and necrosis and the release of DAMPs such as HMGB1, S100A6, S100A8, and HSP70. In this way, DAMPs may modulate the inflammatory response after exposure to cigarette smoke [10–13].

However, the main immunogenic cell death pathway induced by chronic cigarette smoke exposure is unclear.

Myeloid-derived suppressor cells (MDSCs) are the diverse population of cells in the different stage of maturation which are produced in the bone marrow. In mice, they are defined as Gr-1^+^CD11b^+^ cells, which comprise pathologically activated CD11b^+^Ly6CloLy6G^+^ immature granulocytes and CD11b^+^Ly6ChiLy6G^−^ monocytes. In the microenvironment, they mainly suppress T cell function. These cells not only inhibit the proliferation of T lymphocytes but also induce the accumulation of suppressive regulatory T lymphocytes (Treg Foxp3^+^). Moreover, the stimulation of MDSCs with IFN-*γ* led to increased production of IL-10 and TGF-*β*—the most potent known immunosuppressive cytokines [14]. MDSC may also be involved in the metabolism of L-arginine by arginase-1 or inducible nitric oxide synthase [15, 16]. Through some different immunological mechanisms, MDSCs promote tumor progression [16–18]. In the course of COPD, cigarette smoking enhances the number and activity of circulating MDSCs. However, this phenomenon is not observed in smokers with normal lung function. The MDSC activation in COPD is associated with the loss of the T-cell receptor *ζ* chain expression [19]. In addition, after four months of cigarette smoke exposure, the number of MDSCs was elevated in the bone marrow, spleens, and lungs, while this was accompanied by a decreased amount of pulmonary dendritic cells (DCs) [20]. Our previous study results confirm that MDSCs and DAMPs occur in the lung microenvironment and PB in patients with COPD. Smoking history may not affect the number of MDSCs. These cells can be involved in the local inflammatory process in COPD, independently from smoking [21]. On the contrary, DAMPs may also be included in local and systemic inflammatory process in COPD. Concentrations of most of these proteins may be uninfluenced by smoking history, and only a part of the tested DAMPs correlated with clinical parameters. Only a part of triggering DAMPs may influence COPD clinical progression [22].

The aim of the current study was to evaluate the presence of MDSCs and DAMPs in the lung environment using BALF and attempt to determine their mutual relationship in the course of COPD.

## 2. Patients and Methods

### 2.1. Study Population

A study was performed in the Department of Pulmonology, Allergology and Pulmonary Oncology, Poznan University of Medical Sciences, Poland. 30 patients (24 males, 6 females) diagnosed with COPD—according to GOLD 2010 criteria [1] with a relevant history confirmed with a postbronchodilator FEV1/FVC ratio < 0.7—were included in the study. All patients were >40 years old, current and former smokers. Subjects with other pulmonary disorders, such as asthma, tuberculosis, pulmonary thromboembolism, or interstitial pulmonary lesions, and with contraindications to the performance of fiberoptic bronchoscopy or pulmonary function tests were excluded.

The research was carried out in accordance with the protocol approved by the Ethics Committee of the Poznan University of Medical Sciences. All patients gave their written informed consent to participate in the study.

### 2.2. Pulmonary Function Tests

Using a Master Screen Body/Diffusion Jaeger device, spirometry and body plethysmography were performed, and the diffusion capacity of carbon monoxide (DLCO) was measured, by an experienced technician. Spirometry was performed 15–30 min after the inhalation of short-acting bronchodilator. The results were shown as % predicted. The airflow limitation was defined as postbronchodilator FEV1/FVC ratio < 0.70 [1].

### 2.3. Blood Samples

For cytometric immunophenotyping, peripheral blood samples were collected into tubes with EDTA (9 ml). Blood samples for soluble analyte assessment were collected into the tube without anticoagulant (9 ml) and centrifuged at 2500 rpm for 10 min at 4°C. The obtained serum samples were frozen immediately at −70°C for the next investigations.

### 2.4. Fiberoptic Bronchoscopy and BALF

The patients undergoing routine fiberoptic bronchoscopy for diagnostic purposes were enrolled into the study. BAL fluid samples were collected according to international guidelines criteria [23, 24]. The bronchoscope was wedged in the segmental or subsegmental bronchus of the middle lobe. The bronchus was lavaged with 50 ml aliquots of the sterile saline solution at a temperature of 37°C, and then, the fluid was aspirated. Furthermore, two 50 ml aliquots of saline solution were instilled and aspirated in the same way [25, 26].

### 2.5. Immunophenotypic Assessment

**Figure 1:**
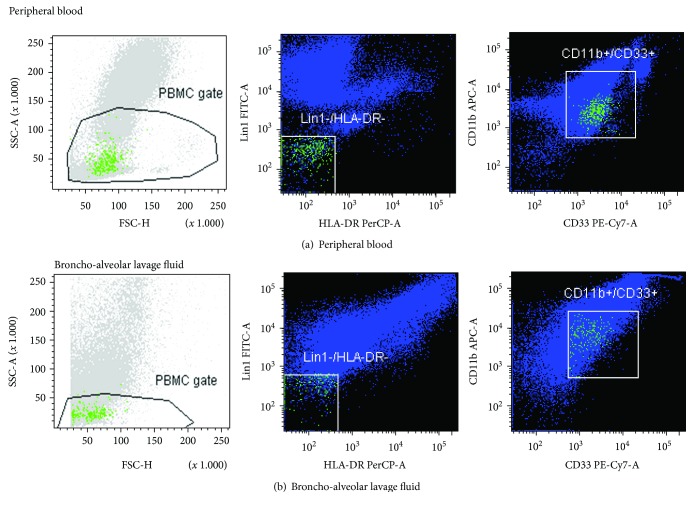
Evaluation of MDSC immunophenotype using a flow cytometer. Scattergrams showing MDSC cells in peripheral blood (a) and in BALF (b). The MDSC immunophenotypic pattern was defined as Lin-1^neg/low^/HLA-DR^neg/low^/CD33^pos^/CD11b^pos^.

Using a flow cytometer, fresh unfixed cells from BALF and peripheral blood were immunophenotyped. The estimation of antigenic determinants, which are characteristic for MDSC populations, was performed using monoclonal antibodies directly conjugated with fluorochromes (Figure 1). MDSCs were defined as cells with immunophenotype SSC^low^/Lin-1^neg^/HLA-DR^neg/low^/CD11^+^/CD33^+^/CD45^+^ [21]. The samples for cytofluorimetric analyses were prepared in the following manner. The initial preparation of BALF samples comprised sterile filtration to remove any mucus, blood clots, and tissue fragments. Cells were then concentrated through centrifugation for 10 min at 1800 rpm. Supernatants were removed, frozen, and stored for later evaluation of the concentration of DAMPs. Antibodies were added to the cell pellets from BALF as well as to peripheral blood samples: 5 *μ*l of each antibody per 2 × 10^5^–1 × 10^6^ of cells in a sample. Negative controls were samples without added antibodies. The following antibodies were used: anti-Human Lineage Cocktail 1 (Lin-1) FITC (cocktail of antibodies against CD3, CD14, CD16, CD19, CD20, and CD56; BD Biosciences), anti-HLA-DR PerCP (clone: L243; BD Biosciences), anti-CD11b APC (clone: ICRF44; BD Biosciences), anti-CD33 PE-Cy™7 (clone: P67.6; BD Biosciences), and anti-CD45 APC-Cy™7 (clone: 2D1; BD Biosciences). Cells were incubated with antibodies for 15 min in the dark. Erythrocyte residues studied were lysed using 2 ml of lysing solution (BD Biosciences). By adding PBS solution after 10 min, lysis was stopped. Finally, all lysed residues, morphotic particles, and soluble proteins were washed out by double centrifugation for 5 min at 1500 rpm. Using a FACS Canto flow cytometer (BD Biosciences, San Jose, CA, USA), appropriately stained cells were analyzed, and the obtained results were processed via FACS Diva software (BD Biosciences, San Jose, CA, USA). Up to 50,000 events of each sample were collected. The percentage of positive cells was assessed [21].

### 2.6. DAMP Concentration Assessment

Serum samples and BALF supernatants were collected before analyses and stored at −80°C. Frozen samples were thawed only once, directly before DAMP concentration assessment. All studied molecules were evaluated in both serum and BALF supernatant samples. Using kits produced by USCN (Wuhan, China), concentrations of defensin (DEF2) (pg/ml) and heat shock protein-27 (HSP27) (ng/ml) were estimated. Surfactant protein A (SP-A) (ng/ml) was assessed using BioVendor kit (Brno, Czech Republic) and hyaluronic acid (HA) (ng/ml) by TECO medical kit (Sissach, Switzerland). All tests were conducted following manufacturers' instructions. Serum samples were diluted with dilution buffer in appropriate ratios: 1 : 10 for DEF2; 1 : 10 for SP-A; 1 : 50 for HA, and additionally, BALF samples for the evaluation of SP-A were diluted in ratio 1 : 100. The measurements were made according to calibration curves. Reactions were stopped with H_2_SO_4_, and the absorbance of colored products was measured at 450 nm using Multiscan Bichromatic (Labsystems) microtitration reader.

There is no standard range of these molecules, and the amount of BALF collected was variable. Therefore, using Bradford assay, the values of DAMPs were corrected for total protein concentration [27].

### 2.7. Statistical Analysis

Using the Statistica 10.0 program, statistical analyses were performed. The Shapiro-Wilk test was used to assess the normality of data distribution and confirmed that all parameters were normally distributed. Results were shown as mean ± standard errors of the mean and median ± standard deviation (SD). We used the Pearson correlation coefficient to assess the presence of a relationship between % of MDSCs in BALF and peripheral blood and DAMP concentration. A *P* value less than 0.05 was considered statistically significant.

## 3. Results

### 3.1. Subject Characteristics

30 COPD patients were enrolled to the study. All patients were current or former smokers with mean smoking history 35.67 ± 4.07 pack years. The mean age of the study group was 66.60 ± 1.53 yrs. The majority of patients who participated in the study belonged to GOLD 2010 stages II and III (FEV1 49.75 ± 3.77% predicted). 29 subjects were assessed using BODE index with mean 3.79 ± 0.49 points. Detailed characteristics of the study population are shown in Table 1.

### 3.2. Soluble Mediator Concentrations

DEF, HSP27, SP-A, and HA were detected and corrected by total protein (TP) in all BAL fluid and serum samples (Tables 2 and 3). The concentrations of all soluble mediators were higher in BAL fluid than in serum.

### 3.3. The Percentage of Myeloid-Derived Suppressor Cells

The percentage of myeloid-derived suppressor cells among all leukocytes and among mononuclear cells in bronchoalveolar lavage fluid (BALF) and peripheral blood was done in whole assessed subject and was lower in BALF. This data was presented in Table 4.

### 3.4. Soluble Mediators vs. MDSCs

**Figure 2:**
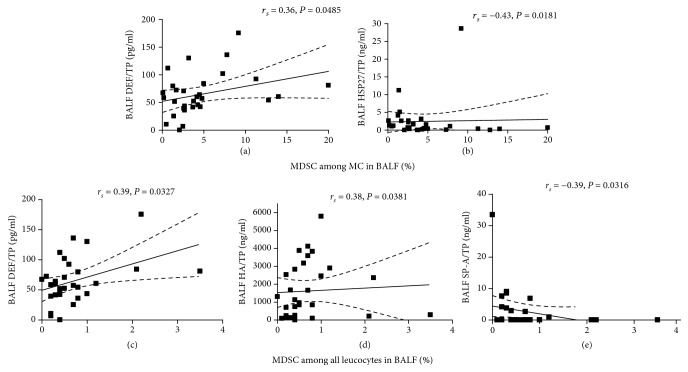
Correlations observed in bronchoalveolar lavage fluid (BALF) between percentage of myeloid-derived suppressor cells (MDSCs) among mononuclear cells (MCs) and defensin (DEF/TP) (a) and heat shock protein-27 (HSP-27) (b). Correlations observed in bronchoalveolar lavage fluid (BALF) between percentage of myeloid-derived suppressor cells (MDSCs) among all leukocytes and defensin (DEF) (c); hyaluronic acid (HA) (d); and surfactant protein A (SP-A) (e). All concentration data were corrected by total protein.

The analysis of bronchoalveolar lavage fluid in the entire group revealed the significant correlation between the percentage of MDSCs among MC and DEF/TP (pg/ml) (*r*_*s*_ = 0.36, *P* = 0.0485) and HSP27/TP (ng/ml) (*r*_*s*_ = −0.43, *P* = 0.0181) (see Figures 2(a) and 2(b)).

We also revealed a significant correlation with BALF DEF/TP (pg/ml) (*r*_*s*_ = 0.39, *P* = 0.0327) (see Figure 2(c)), BALF HA/TP (ng/ml) (*r*_*s*_ = 0.38, *P* = 0.0381) (see Figure 2(d)), and BALF SP-A/TP (ng/ml) (*r*_*s*_ = −0.39, *P* = 0.0316) (see Figure 2(e)), assessing the percentage of MDSCs among all leukocytes.

We did not find any significant dependencies between DAMPs and MDSCs in peripheral blood.

## 4. Discussion

Myeloid-derived suppressor cells (MDSCs) are a heterogeneous population comprising immature macrophages, granulocytes, dendritic cells, and other cells in the early stages of differentiation with strong immunosuppressive activity [16]. Chronic inflammation accompanying various diseases can trigger the release of cytokines and other intercellular mediators that lead to disturbances in the maturation of cells in the bone marrow and the formation of a population of MDSCs with their potentially immunosuppressive function [28]. The expression of proinflammatory cytokines may be activated by pattern recognition receptors (PRRs) [29]. Binding of ligands to TLRs plays an essential role in innate and adaptive immunity in the course of tissue damage [30]. Ligation of DAMPs with TLRs requires the presence of other molecules—most often proteins such as HSPs, S100, HMGB1, versican, fibronectin, hyaluronic acid, and surfactant proteins [31].

Our previous studies confirmed that MDSCs and DAMPs may be involved in the local inflammation in COPD and may aggravate inflammation together with the severity of bronchoconstriction [21, 22]. In the current study, we attempted to find the relations among MDSCs and DAMPs in the lower airways, where the inflammatory process begins and persists, and in the serum, as the sign of the systemic inflammation.

The assessed DAMPs, SP-A, HSP-27, Def, and HA, correlated with MDSCs in BALF in the conducted study. We did not find any dependencies between DAMPs and MDSCs in the blood. These particles independently may take part in the local, nonsystemic inflammatory process in COPD. This hypothesis may be supported by the fact that the observed correlation between MDSCs and chosen protein concentration was always assessed as corrected total protein concentration.

Surfactant protein A (SP-A) is involved in the maintenance of normal lung structure [32]. It is also essential for pulmonary function as it reduces alveolar tension and plays a crucial role in innate immune response and lung homeostasis [33]. Concerning the role of SP-A in COPD, the available literature presents conflicting results. The extensive study conducted by Kobayashi et al. hypothesized that SP-A in plasma and sputum may be considered as an early marker of lung stress reaction/minimal lung injury in smokers, even before any decline of lung function has occurred [34]. Ilumets et al. confirmed that plasma SP-A concentration increases with aging, smoking, and COPD [35]. Mazur et al. evaluated SP-A in induced sputum in COPD. They showed that smoking significantly increases its concentration. Only a few studies were conducted using BALF. They concluded that the concentration of SP-A is lower in smokers/COPD compared to nonsmokers [36, 37]. We achieved a similar result in our previous study, in which we assessed the concentration of SP-A in BALF [22]. In the available literature, we did not find the data concerning the correlation between SP-A and MDSCs in BALF. The current study is the first of its kind undertaking this topic. A lower concentration of SP-A that occurred in the course of COPD may be partially the response for an increased number of MDSCs.

The next interesting protein group belonging to DAMPs is heat shock proteins (HSPs). HSPs are chaperone proteins that are upregulated in various types of physiological and environmental stress conditions. They have been shown to activate TLR2 and TLR4 leading to the release of proinflammatory cytokines [3]. Hulina-Tomašković et al. conducted an in vitro study, in which they explored inflammatory parameters (TLR2, TLR4, and HSP70) after the exposure of bronchial-epithelial cell line to cigarette smoke extract (CSE). They showed that HSP70 modulates inflammatory responses and TLR2, TLR4, and HSP70 gene expression and protects bronchial-epithelial cells against CSE-induced cytotoxicity. They concluded that HSP-70 might be implicated in the development of inflammatory diseases affected by cigarette smoke like COPD [11]. Furthermore, HSPs can activate the maturation and activation of dendritic cells [38]. A few reports have evaluated HSP-27 in COPD. Al Kayal et al. and Ünver et al. have presented significant differences in serum HSP-27 concentration between COPD and healthy controls, with the highest concentration in COPD [39, 40]. Our previous study corresponded with the abovementioned report, confirming the correlation between HSP-27 and PFT in COPD [24]. We did not find the reports concerning cross-talk between HSP-27 and MDSCs in COPD. Only one finding suggested the relationship between HSP-27 and monocytic myeloid suppressor CD14^+^HLA-DR^low/neg^ cells in lymphoma [41]. Therefore, it is difficult to explain the negative correlation between these parameters. A positive relationship should be expected primarily because of the ability of this protein to trigger the IFN-*γ* and IL-10 secretion. On the contrary, MDSCs were elevated in the organs after four months of cigarette smoke exposure, while this was paralleled by decreased pulmonary dendritic cells (DCs) that may be connected with lower HSP-27 concentration [21].

Some of the antimicrobial peptides can also function as DAMPs, and one of the best known are defensins [3]. By binding to the chemokine receptor CCR6, human *β*-defensins attract memory T cells, especially Th 17 subtype, neutrophils, and immature dendritic cells [42]. It also increases the expression of proinflammatory cytokines (e.g., IL-6, IL-8, monocyte chemotactic protein-1, and granulocyte macrophage colony-stimulating factor) and induces necrotic cell death [43]. Pace et al. presented an increased concentration of *β*-defensin-2 in mini-BAL samples of COPD patients compared to nonsymptomatic smokers and healthy volunteers [44]. Our previous study also confirmed the occurrence of *β*-defensin-2 in BALF of COPD patients [21]. On the contrary, Tsoumakidou et al. showed no detectable *β*-defensin-2 in BALF of COPD patients, while it was detectable in control smokers and nonsmokers [45].

The molecules from the extracellular matrix (ECM) may also activate the immune system to danger response [38]. One of these molecules is hyaluronan. There is emerging evidence that HA and its degradation products have an important role in lung pathobiology in COPD [46]. It can activate TLR2 and TL4 downstream NF-k*β* pathways, thereby inducing or maintaining proinflammatory activation of the innate immune system [47, 48]. Unfortunately, there are no data showing the connection between HA and MDSCs in the lung microenvironment. The current study results have given new information about the possible function of HA. It may indirectly influence and increase the number of the BALF MDSC in COPD.

We are aware about some limitations of our study. The first limitation is the lack of a control group. It was impossible to obtain the consent of the Ethics Committee to perform fiberoptic bronchoscopy in healthy subjects, so we designed this study as cross-sectional. The second limitation is the difficulty to define the immunophenotype of MDSCs in humans. These cells are positive for myeloid-lineage markers, CD11b and CD33, but do not express HLA-DR molecules, and they are negative for markers characteristic of lymphoid cells. Recently, MDSCs were subdivided into monocytic (CD14^+^/HLA-DR^−^) and granulocytic (CD15^+^/HLA-DR^−^) MDSCs. In the presented study, MDSCs were defined as cells belonging to leukocytes (CD45^+^), with low granularity (SSC^low^), and express the following immunophenotype: CD11b^+^/CD33^+^/HLA-DR^−^ [49].

## 5. Conclusions

In conclusion, the study confirms that MDSCs and DAMPs occur in the COPD lung microenvironment. Our study is the first to provide information about the relationships between MDSCs and DAMPs in human BALF. Significant correlations between them found in BALF but not in serum indicate that their interactions may influence the local, but not systemic, inflammation in the course of COPD. Moreover, the current study confirms the complex inflammatory pathway in COPD.

## Figures and Tables

**Table 1:** Characteristics of the study population.

	*N*	Mean ± SEM	Median ± SD	Min	Max
Age (years)	30	66.60 ± 1.53	67.5 ± 8.38	52	83
FEV1 (% pred)	30	49.75 ± 3.77	49.5 ± 20.64	22.1	93.2
FVC (% pred)	30	70.87 ± 3.57	67.7 ± 19.54	39.8	112.1
RV (% pred)	29	205.74 ± 14.74	200.1 ± 79.4	28.4	423.8
TLC (% pred)	29	120.53 ± 4.82	117.4 ± 26.0	67.2	188.5
RV/TLC (%)	29	63.12 ± 2.78	66.94 ± 15.0	16.12	87.41
SaO2 (%)	30	93.95 ± 0.35	94.1 ± 1.98	90.4	96.6
PaO2 (mmHg)	29	67.84 ± 1.41	66.9 ± 7.61	57.7	80.5
6MWT (m)	30	326.33 ± 24.09	360.0 ± 131.9	60	545
BODE (1-10)	29	3.79 ± 0.49	3 ± 2.64	0	9
Smoking (pack years)	27	35.67 ± 4.07	30.0 ± 21.16	4	98

Abbreviations: 6MWT: 6-minute walking test; BODE index: B—BMI, O—obstruction, D—dyspnea, E—exercise; FEV1: forced expiratory volume in one second; FVC: forced vital capacity; PaO2: arterial oxygen pressure; RV: residual volume; SaO2: arterial oxygen saturation; TLC: total lung capacity.

**Table 2:** Concentrations of soluble mediators corrected by TP in BAL fluid.

	*N*	Mean ± SEM	Median ± SD	Min	Max
BALF DEF/TP (pg/ml)	30	66.01 ± 7.03	59.30 ± 38.48	0.16	175.4
BALF HSP27/TP (ng/ml)	30	2.49 ± 0.99	0.88 ± 5.412	0	28.61
BALF SP-A/TP (ng/ml)	30	2.66 ± 1.18	0 ± 6.47	0	37.47
BALF HA/TP (ng/ml)	30	1626.63 ± 283.92	1040.13 ± 1555.12	0.77	5789.8

Abbreviations: BALF: bronchoalveolar lavage fluid; DEF: defensin; HA: hyaluronic acid; HSP27: heat shock protein-27; SP-A: surfactant protein A; TP: total protein.

**Table 3:** Concentrations of soluble mediators corrected by TP in serum.

	*N*	Mean ± SEM	Median ± SD	Min	Max
Serum DEF/TP (pg/ml)	22	2.38 ± 0.48	1.52 ± 2.25	0.92	10.33
Serum HSP27/TP (ng/ml)	22	0.00 ± 0.00	0.00 ± 0.00	0	0.01
Serum SP-A/TP (ng/ml)	22	0.02 ± 0.00	0.02 ± 0.01	0.01	0.07
Serum HA/TP (ng/ml)	22	0.54 ± 0.11	0.37 ± 0.53	0	1.78

Abbreviations: DEF: defensin; HA: hyaluronic acid; HSP27: heat shock protein-27; SP-A: surfactant protein A; TP: total protein.

**Table 4:** The percentage of MDSCs among all leukocytes and MC in BALF and peripheral blood.

	*N*	Mean ± SEM	Median ± SD	Min	Max
BALF MDSCs (%)	30	0.71 ± 0.13	0.48 ± 0.72	0.04	3.45
BALF MDSCs (% of MC)	30	4.77 ± 0.84	3.43 ± 4.61	0.12	20.01
Blood MDSCs (%)	30	4.33 ± 0.68	2.98 ± 3.72	1.39	16.02
Blood MDSCs (% of MC)	30	12.73 ± 1.54	9.99 ± 8.45	3.49	36.36

Abbreviations: BALF: bronchoalveolar lavage fluid; MC: mononuclear cells; MDSCs: myeloid-derived suppressor cells.

## Data Availability

All necessary data remains in the possession of the authors of the article.

## References

[1] Global Initiative for Chronic Obstructive Lung Disease (GOLD) (2010). *Global strategy for the diagnosis, management, and prevention of chronic obstructive pulmonary disease*.

[2] Vaguliene N., Zemaitis M., Lavinskiene S., Miliauskas S., Sakalauskas R. (2013). Local and systemic neutrophilic inflammation in patients with lung cancer and chronic obstructive pulmonary disease. *BMC Immunology*.

[3] Pouwels S. D., Heijink I. H., ten Hacken N. H. (2014). DAMPs activating innate and adaptive immune responses in COPD. *Mucosal Immunology*.

[4] Matzinger P. (1994). Tolerance, danger, and the extended family. *Annual Review of Immunology*.

[5] Huang J., Xie Y., Sun X. (2015). DAMPs, ageing, and cancer: the ‘DAMP hypothesis’. *Ageing Research Reviews*.

[6] Łagiedo M., Sikora J., Kaczmarek M. (2015). Damage-associated molecular patterns in the course of lung cancer – a review. *Scandinavian Journal of Immunology*.

[7] Land W. G. (2015). The role of damage-associated molecular patterns (DAMPs) in human diseases part II: DAMPs as diagnostics, prognostics and therapeutics in clinical medicine. *Sultan Qaboos University Medical Journal*.

[8] Boyapati R. K., Rossi A. G., Satsangi J., Ho G. T. (2016). Gut mucosal DAMPs in IBD: from mechanisms to therapeutic implications. *Mucosal Immunology*.

[9] Pouwels S. D., Zijlstra G. J., van der Toorn M. (2016). Cigarette smoke-induced necroptosis and DAMP release trigger neutrophilic airway inflammation in mice. *American Journal of Physiology-Lung Cellular and Molecular Physiology*.

[10] Faiz A., Heijink I. H., Vermeulen C. J. (2018). Cigarette smoke exposure decreases *CFLAR* expression in the bronchial epithelium, augmenting susceptibility for lung epithelial cell death and DAMP release. *Scientific Reports*.

[11] Hulina-Tomašković A., Rajković M. G., Somborac-Bačura A., Čeri A., Dabelić S., Rumora L. (2018). Extracellular Hsp70 modulates the inflammatory response of cigarette smoke extract in NCI-H292 cells. *Experimental Physiology*.

[12] Hulina-Tomašković A., Heijink I. H., Jonker M. R., Somborac-Bačura A., Grdić Rajković M., Rumora L. (2019). Pro-inflammatory effects of extracellular Hsp70 and cigarette smoke in primary airway epithelial cells from COPD patients. *Biochimie*.

[13] Lee H., Lee J., Hong S. H., Rahman I., Yang S. R. (2018). Inhibition of RAGE attenuates cigarette smoke-induced lung epithelial cell damage via RAGE-mediated Nrf2/DAMP signaling. *Frontiers in Pharmacology*.

[14] Gabrilovich D. I., Nagaraj S. (2009). Myeloid-derived suppressor cells as regulators of the immune system. *Nature Reviews Immunology*.

[15] Peranzoni E., Zilio S., Marigo I. (2010). Myeloid-derived suppressor cell heterogeneity and subset definition. *Current Opinion in Immunology*.

[16] Talmadge J. E., Gabrilovich D. I. (2013). History of myeloid-derived suppressor cells. *Nature Reviews Cancer*.

[17] Lindau D., Gielen P., Kroesen M., Wesseling P., Adema G. J. (2013). The immunosuppressive tumour network: myeloid-derived suppressor cells, regulatory T cells and natural killer T cells. *Immunology*.

[18] Keskinov A. A., Shurin M. R. (2015). Myeloid regulatory cells in tumor spreading and metastasis. *Immunobiology*.

[19] Scrimini S., Pons J., Agustí A. (2013). Differential effects of smoking and COPD upon circulating myeloid derived suppressor cells. *Respiratory Medicine*.

[20] Ortiz M. L., Lu L., Ramachandran I., Gabrilovich D. I. (2014). Myeloid-derived suppressor cells in the development of lung cancer. *Cancer Immunology Research*.

[21] Brajer-Luftmann B., Nowicka A., Kaczmarek M. (2016). Myeloid-derived suppressor cells in bronchoalveolar lavage fluid in patients with chronic obstructive pulmonary disease. *Polish Archives of Internal Medicine*.

[22] Brajer-Luftmann B., Nowicka A., Kaczmarek M., Pokorski M. (2018). Molecules of damage-associated patterns in bronchoalveolar lavage fluid and serum in chronic obstructive pulmonary disease. *Respiratory Ailments in Context*.

[23] Klech H. (1989). Technical recomendations and guidelines for bronchoalveolar lavage (BAL). *European Respiratory Journal*.

[24] Meyer C., Cagnon L., Costa-Nunes C. M. (2014). Frequencies of circulating MDSC correlate with clinical outcome of melanoma patients treated with ipilimumab. *Cancer Immunology, Immunotherapy*.

[25] Kelsen S. G., Mardini I. A., Zhou S., Benovic J. L., Higgins N. C. (1992). A technique to harvest viable tracheobronchial epithelial cells from living human donors. *American Journal of Respiratory Cell and Molecular Biology*.

[26] Redakcja PiAP (2011). Recommendation of the Polish Respiratory Society for bronchoalveolar lavage (BAL) sampling, processing and analysis methods. *Pneumonologia i Alergologia Polska*.

[27] Bradford M. M. (1976). A rapid and sensitive method for the quantitation of microgram quantities of protein utilizing the principle of protein-dye binding. *Analytical Biochemistry*.

[28] Gabrilovich D. I. (2017). Myeloid-derived suppressor cells. *Cancer Immunology Research*.

[29] Hirsiger S., Simmen H. P., Werner C. M. L., Wanner G. A., Rittirsch D. (2012). Danger signals activating the immune response after trauma. *Mediators of Inflammation*.

[30] Janeway C. A., Medzhitov R. (2002). Innate immune recognition. *Annual Review of Immunology*.

[31] Piccinini A. M., Midwood K. S. (2010). DAMPening inflammation by modulating TLR signalling. *Mediators of Inflammation*.

[32] Haczku A. (2008). Protective role of the lung collectins surfactant protein A and surfactant protein D in airway inflammation. *The Journal of Allergy and Clinical Immunology*.

[33] Nathan N., Taytard J., Duquesnoy P. (2016). Surfactant protein A: a key player in lung homeostasis. *The International Journal of Biochemistry & Cell Biology*.

[34] Kobayashi H., Kanoh S., Motoyoshi K. (2008). Serum surfactant protein-A, but not surfactant protein-D or KL-6, can predict preclinical lung damage induced by smoking. *Biomarkers*.

[35] Ilumets H., Mazur W., Toljamo T. (2011). Ageing and smoking contribute to plasma surfactant proteins and protease imbalance with correlations to airway obstruction. *BMC Pulmonary Medicine*.

[36] Honda Y., Takahashi H., Kuroki Y., Akino T., Abe S. (1996). Decreased contents of surfactant proteins A and D in BAL fluids of healthy smokers. *Chest*.

[37] Betsuyaku T., Kuroki Y., Nagai K., Nasuhara Y., Nishimura M. (2004). Effects of ageing and smoking on SP-A and SP-D levels in bronchoalveolar lavage fluid. *European Respiratory Journal*.

[38] Asea A., Rehli M., Kabingu E. (2002). Novel signal transduction pathway utilized by extracellular HSP70: role of toll-like receptor (TLR) 2 and TLR4. *Journal of Biological Chemistry*.

[39] Al Kayal H., Bass H. A., El Sabbah A. (2015). Can HSP27 and HSP70 be used as biomarkers for chronic obstructive pulmonary disease diagnosis?. *European Respiratory Journal*.

[40] Unver R., Deveci F., Kirkil G., Telo S., Kaman D., Kuluozturk M. (2016). Serum heat shock protein levels and the relationship of heat shock proteins with various parameters in chronic obstructive pulmonary disease patients. *Turkish Thoracic Journal*.

[41] Zhang Z. (. J.)., Bulur P. A., Dogan A., Gastineau D. A., Dietz A. B., Lin Y. (2015). Immune independent crosstalk between lymphoma and myeloid suppressor CD14^+^HLA-DR^low/neg^ monocytes mediates chemotherapy resistance. *OncoImmunology*.

[42] Rosin D. L., Okusa M. D. (2011). Dangers within: DAMP responses to damage and cell death in kidney disease. *Journal of the American Society of Nephrology*.

[43] Van Wetering S., Mannesse-Lazeroms S. P. G., Van Sterkenburg M. A. J. A., Hiemstra P. S. (2002). Neutrophil defensins stimulate the release of cytokines by airway epithelial cells: modulation by dexamethasone. *Inflammation Research*.

[44] Pace E., Giarratano A., Ferraro M. (2011). TLR4 upregulation underpins airway neutrophilia in smokers with chronic obstructive pulmonary disease and acute respiratory failure. *Human Immunology*.

[45] Tsoumakidou M., Bouloukaki I., Thimaki K., Tzanakis N., Siafakas N. M. (2010). Innate immunity proteins in chronic obstructive pulmonary disease and idiopathic pulmonary fibrosis. *Experimental Lung Research*.

[46] Garantziotis S., Brezina M., Castelnuovo P., Drago L. (2016). The role of hyaluronan in the pathobiology and treatment of respiratory disease. *American Journal of Physiology-Lung Cellular and Molecular Physiology*.

[47] Papakonstantinou E., Roth M., Klagas I., Karakiulakis G., Tamm M., Stolz D. (2015). COPD exacerbations are associated with proinflammatory degradation of hyaluronic acid. *Chest*.

[48] Turino G. M., Ma S., Lin Y. Y., Cantor J. O. (2018). The therapeutic potential of hyaluronan in COPD. *Chest*.

[49] Ohki S., Shibata M., Gonda K. (2012). Circulating myeloid-derived suppressor cells are increased and correlate to immune suppression, inflammation and hypoproteinemia in patients with cancer. *Oncology Reports*.

